# Cost effective dye sensitized solar cell based on novel Cu polypyrrole multiwall carbon nanotubes nanocomposites counter electrode

**DOI:** 10.1038/s41598-021-94404-0

**Published:** 2021-07-21

**Authors:** Shaista Rafique, Imran Rashid, Rehana Sharif

**Affiliations:** 1 Department of Physics, Government College Women University, Faisalabad, Pakistan; 2 Electrical Engineering Department, The University of Lahore, Islamabad, Pakistan; 3 Department of Physics, University of Engineering and Technology Lahore, Lahore, Pakistan

**Keywords:** Electronics, photonics and device physics, Photonic devices, Nanoscience and technology, Nanoscale devices

## Abstract

In order to replace Pt CE in dye sensitized solar cell (DSSC) with simple and low cost, copper polypyyrol functionalized multiwall carbon nanotubes (Cu-PPy-FWCNTS) nanocomposite CE was fabricated by two step electrodeposition method on the stainless-steel substrate. The surface morphology, electrical conductivity, electrochemical properties of Cu-PPy-FWCNTS nanocomposite CE electrodes were observed by using verity of techniques such as scanning electron microscopy, a four-probe method and electrochemical workstation. The Fourier transform infrared (FTIR) spectroscopy confirms the presence of FMWCNTS into PPy-FMWCNTS nanocomposite and XRD analysis verified the Cu nanostructures had come into being. The cyclic voltammogram and Tafel polarization measurement demonstrated that solution processed Cu-PPy-FWCNTS nanocomposites CE had smaller charge transfer resistance R_ct_ (4.31 Ω cm^2^) and higher electrocatalytic performance for I_3_^−^/I^−^ redox solution. Finally, the photovoltaic efficiency of DSSC assembled with Cu-PPy-FWCNTS nanocomposite CE and Platinized CE were compared. The results revealed that the photovoltaic efficiency of DSSC with Cu-PPy-FWCNTS nanocomposites CE reached (7.1%), which is superior to Platinized CE (6.4%). The higher photovoltaic efficiency of the Cu-PPy-FMWCNTS film is due to copper nanostructures that lead to higher cathodic current density (2.35 mA/cm^2^). The simple fabrication method, excellent electrocatalytic and photovoltaic properties permit the Cu-PPy-FWCNTS nanocomposites credible alternative CE to save the cost of DSSC.

## Introduction

DSSCs have received considerable scientific interest owing to their merits on easy fabrication process, cost effectiveness and maximum photovoltaic efficiency of 12.3% than traditional silicon solar cell^1–6^. The standard DSSCs are made up of dye sensitized Titanium Dioxide (TiO_2_) working electrode, I_3_^−^/I^−^ redox electrolyte and a Counter Electrode^7–9^. The counter electrode is one of the crucial and indispensable component of DSSc that plays a vital role to transport the electron from outer circuit to redox electrolyte^10,11^. The standard DSSC comprised of platinum counter electrode because of their good electric conductivity and excellent electrocatalytic activity toward triiodide reduction, however, its high price, rarity on earth and usage of complex vacuum system are the major bottlenecks in the large-scale production of DSSCs^12–14^. Therefore, scientists are struggling to develop some cost-effective catalytic materials as an alternative to platinum counter electrode. Numerous verities of cost-effective materials such as various carbonous materials, conducting polymers, metal oxides and transition metal carbides have been reported to substitute the platinum counter electrode CE^15–21^.

Recently, carbonaceous materials such as carbon black, graphite carbon nanotubes, graphene, and composite of previous materials have been reported as alternative catalytic materials to Pt CEs for large scale production of DSSC^22–28^. The composite film of platinum nanoparticles (PtNP)/PANI deposited on poly(ethylene naphthalate) film have been investigated as an alternative CE and exhibit noticeable electrocatalytic activity for the reduction of triiodide due to combined effect of both PANI and PtNP^29^. Some polymer-based materials have been intensively used as hole conductor electrolyte additive and plastic substrate in DSSCs. The main advantage of using polymeric materials is the building of flexible and low-cost solar cells, which are beneficial for transportation in complex environments as compared with the rigid cells^30–35^.

Recently cobalt triphosphide *CoP*_3_ on carbon paper was successfully synthesized by using a two step method and serviced as CE in DSSC. The synergistic effect of carbon paper and cobalt triphosphide can exhibit greatly improved photovoltaic efficiency in DSSC^36^. Composite film of *PPy*/*srTiO*_2_ nano particles synthesized by oxidative polymerization technique showed increased surface area, good electrocatalytic performance towards triodide reduction and exhibit 2.52%electrical conversion efficiency^37^. A new strategy have been designed to developed a composite of cobalt phosphide and cobalt molybdenum phosphorus *CoP*/*CoMoP*_2_ on carbon paper via a low temperature phosphating technique and hydrothermal method. The DSSC assembled with composite *CoP*/*CoMoP*_2_ CE delivered a high photovoltaic efficiency of 8.69%^38^. The nitrogen doped mesopours carbon and cobalt phosphide nanoparticles composite COP/NMC CE based DSSC exhibit an impressive power conversion efficiency of 8.53%^39^.

In this research work we used two step electrochemical deposition method for fabrication of inexpensive and simple Cu-PPy-FMWCNTs nanocomposites. The PPy-FMWCNTS nanocomposite film is synthesis on stainless steel substrate by chrono amperometric method and then the cyclic voltammetry is used to modify the obtained film with Cu nanostructures. Both these deposition methods are economical and simple for commercialization of DSSCs. It is expected that Cu-PPy-FMWCNTS nanocomposite CEs exhibit higher electrocatalytic performance and large electrical conductivity due to integrated effect of three constituents and will deliver the improved photovoltaic efficiency.

## Experimental

### Preparation of PPy, PPy-FMWCNTS, Cu-PPy-FMWCNTS nanocomposite CE

Cu-PPy-FMWCNTS nanocomposites films were prepared on the stainless-steel substrate with a simple electrochemical method by functionalizing MWCNTs in a concentrated solution of H_2_SO_4_:HNO_3_ (3:1) and refluxed at 90 °C for one hour according to the previously reported method^40^. Electrochemical synthesis of Cu-PPy-FMWCNTS nanocomposites were performed in a standard three-electrode cell (SS sheet as a working electrode, saturated calomel electrode (SCE) as reference electrode, graphite as counter electrode) using Princeton 263 A electrochemical workstation. The PPy-FMWCNTS nanocomposite film was deposited on stainless steel substrate in an aqueous solution of 10 mg FMWCNTS, 0.2 M PPy, and 0.2 M H_2_SO_4_ at an applied potential of 1.0 V. The Cu was introduced on PPy-FMWCNTS nanocomposites films to form Cu-PPy-FMWCNTS through cyclic voltammetry in an aqueous solution of 0.01MCuSO_4_ and 0.5MH_2_SO_4_ at an applied potential of − 1.0 V.

### Fabrication of DSSCs

The TiO_2_ photoanode was prepared according to the literature^1,41^. In brief a compact TiO_2_ blocking layer was cast on to the FTO substrate by treating it in 50 mM TiCl_4_ isopropanol solution for 30 mints at 70 °C, then sintered at 450 °C for 30 min. Subsequently the TiO_2_ paste was loaded on the compact layer by doctor blade method. After coating the obtained TiO_2_, working electrode was annealed at 450 °C for 30 min. These prepared films were socked in 0.5 mM N719 dye solution in acetonitrile/tert-butanol (1:1 v/v) overnight. The dye socked TiO_2_ photoanode were removed from the dye solution, rinsed with water and dried. Finally, the DSSCs with an active area of 1cm^2^ were assembled by clamping the dye adsorbed TiO_2_ photoanode and CE together and the middest were filled with the drop of liquid electrolyte consisting of anacetonitrile. The DSSCs were wrapped with hot-melt thermoplastic Surlyn. Thermally reduced Pt CE is also used for comparison of photovoltaic efficiency.

### Characterization and measurements

Fourier transform infrared spectrum (FTIR) of PPy, PPy-FMCNTS and Cu-PPy-FMWCNTS nanocomposites CEs was recorded in the range of 500–4000 cm^−1^ using an Infrared Spectrometric Analyzer. The electrical conductivity of the samples was carried out by four probe method. The X-ray diffractogram of the prepared samples were studied by 2001 Bruker-AXS diffractometer using CuKa radiation. The scanning electron microscopy (SEM; JSM-6480LV) was used to characterize the surface morphologies of CEs. Cyclic voltammogram (CV) were recoded with three-electrode arrangements using Princeton 263A electrochemical workstation. Saturated calomel was used as a reference electrode, graphite was used as a counter electrode and PPy, PPy-FMWCNTS, Cu-PPy-FMWCNTS nanocomposite films were used as working electrode. The photovoltaic efficiency of DSSC was obtained with a solar simulator under100 mW/cm^2^.

## Results and discussion

**Figure 1:**
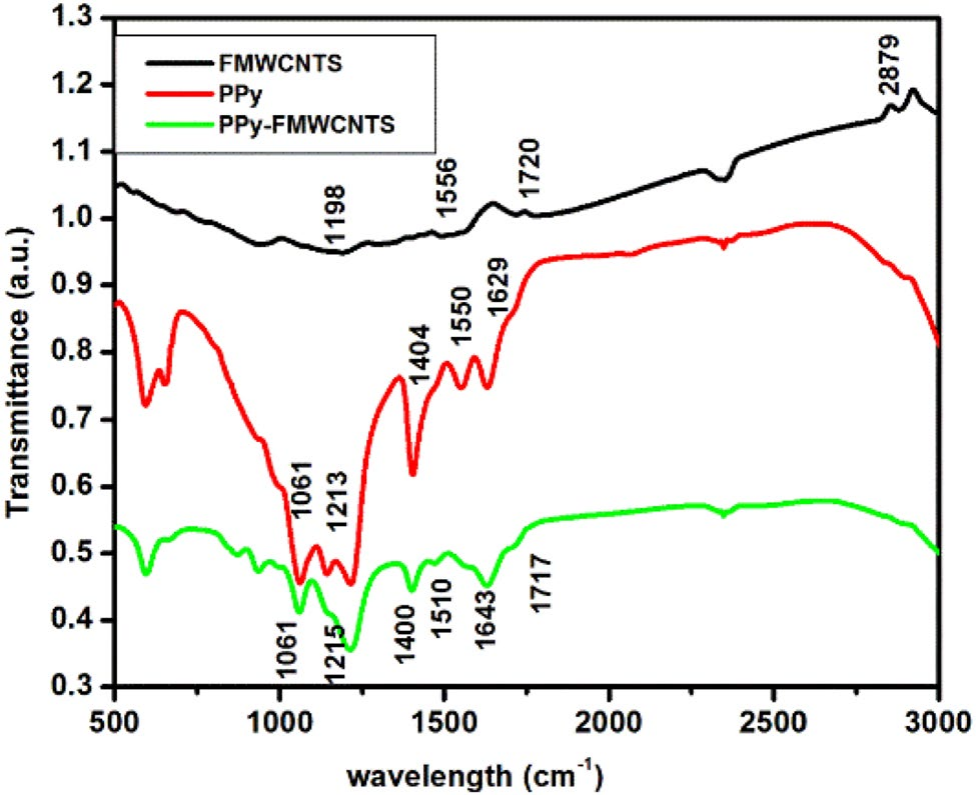
FTIR spectra of FMWCNTS, PPy and PPy-FMWCNTS.

Figure 1 shows the Fourier transform infrared spectrum of FMWCNTS, PPy and PPy-FMWCNTS nanocomposite CEs.

The absorption bands situated at 1556 cm^−1^, 1720 cm^−1^, 1189 cm^−1^ and 2879 cm^−1^ can be viewed in FMCNTS curve. These bands are attributed to stretching of carbon nanotube backbone, C=O stretching of COOH group, C–O–C stretching and –CH_2_– stretching mode respectively, indicating the development of carboxylic groups (–COOH) on the MWCNTS. The characteristic absorption peaks of the pure PPy appeared at 1404 cm^−1^ and 1213 cm^−1^ are indexed to C–N stretching mode of vibration in PPy ring^42–47^. C–N and C–C asymmetric and symmetric ring vibration at 1629 cm^−1^ and 1550 cm^−1^ was also noticed. N–H stretching vibration and C–H in plan deformation appeared at 1061 cm^−1^^48–51^.

In PPy-FMWCNTS nanocomposite curve C–H, C–C and N–H bonds become weaker and C–N bond become stronger. It was observed that the inclusion of FMWCNTS into PPy resulted in an enhanced intensity of some peaks with a little shift. This fact might be attributed to presence of carboxylic group on the nanotube surface that gives the interaction on the different reaction sites of PPy that facilitated the fast electron transportation between PPy and FMWCNT. It was also observed that some peaks were disappeared in PPy-FMWCNTS nanocomposite curve; this was because acid treated MWCNTS were wrapped in PPy thin film^47,49^.

The electrical conductivity of pure PPy, PPy-FMWCNTS and Cu-PPy-FMWCNTS nanocomposites were measured by four probe technique and the obtained results are listed in Table 1.

**Table 1:** Conductivity measurements of PPy, PPy-FMWCNTS and Cu-PPy-FMWCNTS nanocomposites CEs.

Electrodes	Conductivity (S/cm)
PPy	35
PPy-FMWCNTS	250
Cu-PPy-FMWCNTS	280

The value of electrical conductivity is found to be 250 s/cm for PPy-FMWCNTS for nanocomposite where, as the pure PPy has low conductivity of 35 s/cm. The lower value of conductivity of PPy is due to small localization length that is found to be 1.55 nm. In case of FMWCNTS the localization length is around to 10 nm due to presence of large arrangement of π-conjugated structure hence it has high conductivity. Therefore, it is obvious that the introduction of highly conducting FMWCNTS into PPy matrix would increase the average localization length. Moreover, FMWCNTS are considered as a good electron acceptor and PPy is relatively good electron donor. So, there is strong interaction occurred between FMWCNTS and PPy quinoid rings which facilitated the fast movement of the charges between the two components and causes to increase the conductivity of PPy-FMWCNTS nanocomposite film^52,53^. The deposition of Cu nanoparticles over PPy-FMWCNTS provides a unique path for facial electron transport through network assembly and causes an enhancement in electrical conductivity of 280 s/cm for Cu-PPy-FMWCNTS nanocomposite film^54^.

**Figure 2:**
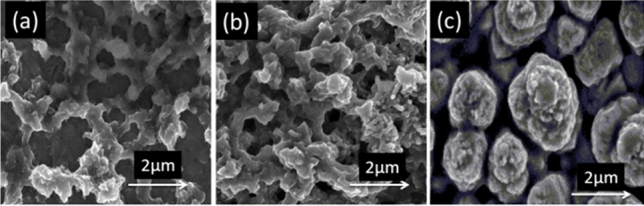
SEM images of (**a**) PPy, (**b**) PPy-FMWCNTS, (**c**) Cu-PPy-FMWCNTS.

Figure 2a–c shows the SEM micrographs of pure PPy, PPy-FMWCNTS and Cu-PPy-FMWCNTS nanocomposites prepared by two step electrodeposition technique on stainless steel substrate. It can be clearly seen that PPy nanoparticles of average diameter of 100 nm are uniformly covered on SS sheet and exhibit a porous structure. It can be observed in Fig. 2b that fiber like FMWCNTS are wrapped with PPy nanoparticles in PPy-FMWCNTSnanocomposite film. This network assembly is helpful for electrical transport between PPy nanoparticle sand FMWCNTS^42,55^. It can also be observed in Fig. 2c that spherical like morphology of Cu nanostructures is homogenously covered on the surface of PPy-FMWCNTS.

These Cu nanostructures appeared as white bright flowers. Moreover, Cu-PPy-FMWCNTS nanocomposite film has porous structure and contributed large surface area to the sufficient adsorption of the electrolyte, which is helpful for the improvement of catalytic activity for I_3_^−^/I^−^ redox solution^54^.

**Figure 3:**
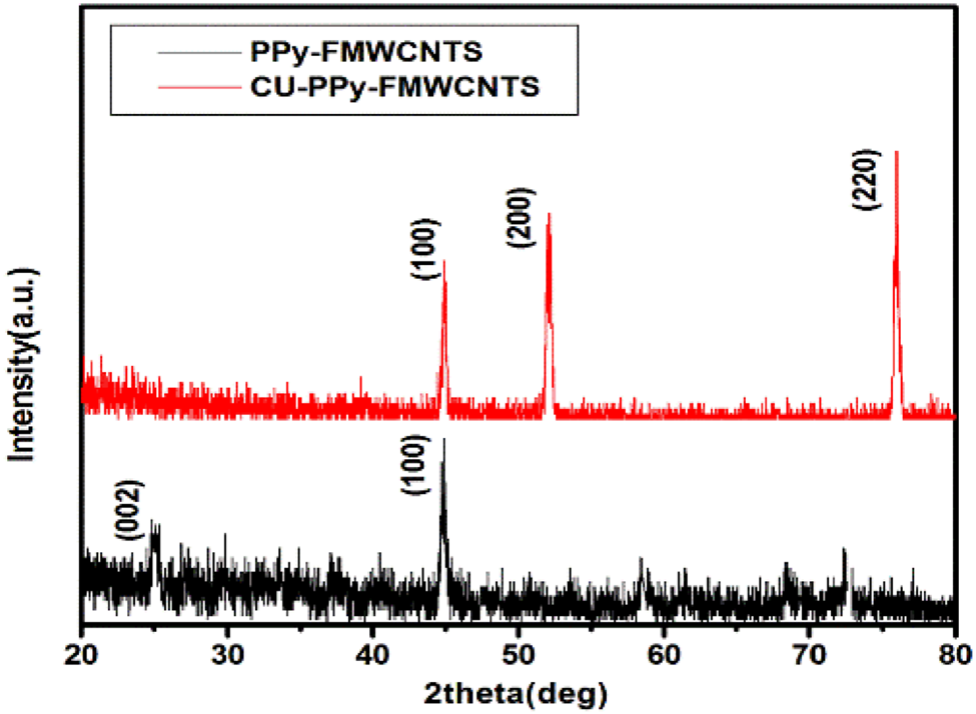
XRD spectra of PPy-FMWCNTs, Cu-PPy-FMWCNTs nanocomposites.

X-ray diffraction pattern was carried out to analyze the crystalline structure of PPy-FMWCNTS and Cu-PPy-FMWCNTS nanocomposite as shown in Fig. 3. In case of PPy-FMWCNTS nanocomposites, two peaks at 2θ = 25°, 43° indicating Bragg’s reflections from (111) and (200) planes respectively^49,56^ which is mainly due to the presence of FMWCNTS. For Cu-PPy-FMWCNTS nanocomposites, the characteristic peaks at 2θ = 25°, 52°, 75° having miller indices (111), (200), and (220) respectively. It is noticed the peak at 2θ = 25°, 43° in Cu-PPy-FMWCNTS nanocomposites film is disappeared. This fact is due to deposition of Cu nanostructures over the PPy-FMWCNTS film. These three diffraction peaks centered at 2θ = 25°, 52°, and 75° represents the formation of metallic Cu nanostructures^54,57^. The presence of Cu nanostructures in Cu-PPy-FMWCNTS nanocomposites film is confirmed by SEM results also.

**Figure 4:**
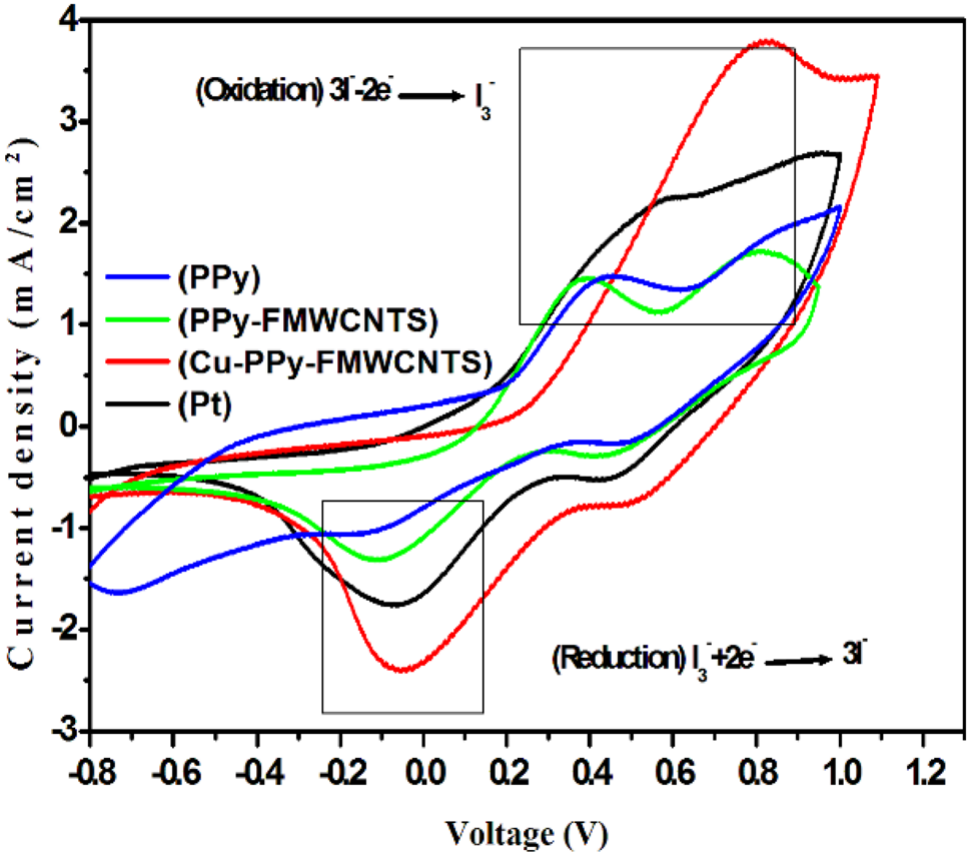
CVs of DSSC based on PPy, PPy-FMWCNTS, and Cu-PPy-FMWCNTS nanocomposites CEs.

Figure 4 shows the I_3_^−^/I^−^ system on the PPy, PPy-FMWCNTS, Cu-PPy-FMWCNTS nanocomposite CEs and Pt electrode in order to analyses the catalytic behavior of CEs. The electrolyte used in CV measurement is acetonitrile solution consisting of 10 mM lithium iodide (LiI), 0.1 M lithium perchlorate (LiClO_4_) as a supporting electrolyte and 1 mM I_2_ as I_3_^−^/I^−^ redox couple with the scan rate of 50 mV/s. In the cyclic voltammograms, two sets of redox peaks were obtained, and the peaks obtained at the positive side are known as anodic peak and the left one is cathodic peak. The anodic peaks in the CV curves refer to the oxidation of iodide to tri-iodide and the cathodic peak is due to the reduction of tri-iodide^58^. All the CEs showed both oxidation and reduction peaks which show that the catalytic performance is comparable to thermally decomposed Ptelectrode. CE is responsible to the reduction of I_3_^−^/I^−^ redox couple, therefore cathodic reaction is considered only. It is observed that the current density for Cu-PPy-FMWCNTS nanocomposite CE is higher than PPy, PPy-FMWCNTS nanocomposite which indicates rapid reaction rate of I_3_^−^/I^−^ on Cu-PPy-FMWCNTS nanocomposite CE and lower charge transfer resistance^27,47,59^. The value of short circuit density J_sc_ (shown in Table 2) follows the order of Cu-PPy-FMWCNTS > Pt > PPy-FMWCNTS > PPy representing the same trend of electrocatalytic activity which could be explained by the reason that the active surface area increases in the same order.

**Table 2:** Cyclic voltammetry and Tafel polymerization measurements of various counter electrodes.

CEs	I_pc_ (mA cm^−2^)	E_pc_ (V)	J_0_ (mA cm^−2^)	R_ct_ (Ω cm^2^)
PPy	1.04	-0.13	2.36	5.43
PPy-FMWCNTS	1.29	-0.11	2.42	5.29
Cu-PPy-FMWCNTS	2.35	-0.57	2.94	4.31
Pt	1.73	-0.06	2.63	4.87

It can be clearly seen from Fig. 4 that the reduction potential of Cu-PPy-FMWCNTS nanocomposite CE is more positive compared to Pt CE indicating the adsorption of liquid electrolyte on to the Cu-PPy-FMWCNTS nanocomposite surface^42^.

**Figure 5:**
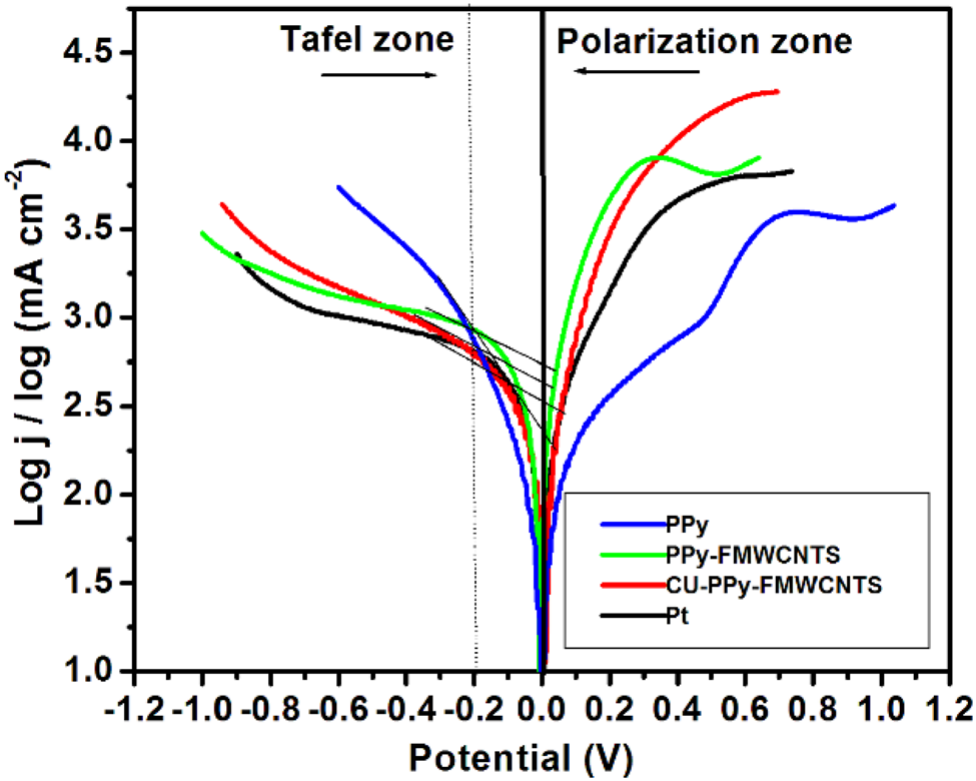
Tafel curves of DSSCs with, PPy, PPy-FMWCNTS and Cu-PPy-FMWCNTS nanocomposites as counter electrodes.

In order to confirm the electrocatalytic activity of the of I_3_^−^/I^−^ redox couple on CEs, Tafel polarization curves were performed in a symmetrical cell containing the same electrolyte that was used in the assembly of DSSCs. In Tafel curve, the logarithm current density (log j) is a linear function of voltage (v) for the reduction of I_3_^−^/I^−^ redox couple, as shown in Fig. 5. Theoretically, the curve at potential less than 0.4v and greater than 0.10v corresponds the Tafel zone^41^. In addition, exchange current density (j_0_) is inversely proportional to charge transfer resistance (R_ct_) according to Eq. (1) and is measured by steep slope of the curve in the Tafel zone^9^.

1 $${\text{R}}_{{{\text{ct}}}} = \frac{{{\text{RT}}}}{{{\text{nFj}}_{0} }}$$

In Eq. (1) J_0_ is the exchange current density, R is the gas constant, T is the absolute temperature,α is the distribution coefficient, F is Faraday’s’ constant, and n (n = 2) is the number of electrons involved in the reaction at the electrode and R_ct_ is the charge transfer resistance at the electrode/electrolyte interface^6,9^.

The slopes of the curves for anodic and cathodic branches in Tafel zone are in the order of Pt > Cu-PPy-FMWCNTS > PPy-FMWCNTS > PPy. A steep slope for anodic and cathodic branches in Tafel zone indicates the presence of higher electrical conductivity and large surface area. This is the key factor for higher electrocatalytic activity. The highest j_o_ is observed for Cu-PPy-FMWCNTS indicating its larger surface area and lower R_ct_ resulting their higher catalytic activity for tri-iodide reduction and improved photovoltaic efficiency^60^.

**Figure 6:**
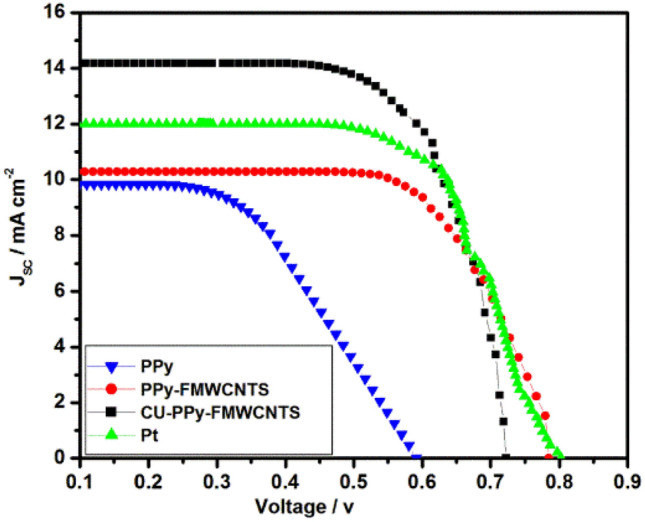
Photocurrent density–voltage curves of DSSCs with, PPy, PPy-FMWCNTS and Cu-PPy-FMWCNTS nanocomposites as CEs.

Figure 6 compares the photocurrent voltage characteristic curve of DSSC based on various CEs and the obtained photovoltaic parameters are listed in Table 3.

**Table 3:** Photovoltaic properties of DSSC with various nanocomposite CEs.

CEs	J_sc_ (mA cm^−2^)	V_oc_ (V)	FF	η (%)
PPy	9.83	0.59	0.525	3.04 ± .02
PPy-FMWCNTS	10.27	0.78	0.682	5.49 ± .05
Cu-PPy-FMWCNTS	14.17	0.72	0.696	7.1 ± .06
Pt	11.99	0.80	.674	6.48 ± .03

The short circuit current density (J_sc_) value of Cu-PPy-FMWCNTS is highest as compared to PPy-FMWCNTS and PPy,This fact might be attributed to large electrical conductivity and homogenously distributed Cu nanostructures, which provides the larger surface areas for I_3_^−^ reduction as indicated in the CV curves^4^. Generally, V_OC_ value of each photovoltaic device depend on the difference between the electronic fermi level in TiO_2_ semiconductor and the formal potential of I_3_^−^/I^−^ redox couple on CE. All the photovoltaic devices fabricated in the present work made up of same type of TiO_2_ photoelectrode and same composition of the liquid electrolyte, Therefore the V_OC_ value of each photovoltaic device is mainly dependent on the electrocatalytic properties of the CEs. It is observed that V_OC_ value of DSSC with Cu-PPy-FMWCNTS CE is highest among all the CEs study in the present work. This might be attributed to reduction potential on Cu-PPy-FMWCNTS CE is more positive than other CEs as shown in Fig. 4^1,61,62^. It is observed in Table 2 that R_ct_ follows the order of PPy > PPy-FMWCNTS > Pt > Cu-PPy-FMWCNTS. Moreover, The DSSC based on Cu-PPy-FMWCNTS CE shows the highest FF (0.696) value than PPy-FMWCNTS and PPy CEs but lower than Pt CE (0.674). This can possibly ascribe to its lower R_ct_ (4.31 Ω cm^2^) than PPy-FMWCNTS (5.29 Ω cm^2^), PPy (5.43 Ω cm^2^) and PtCE (4.87 Ω cm^2^)^42^.

It is obvious from whole results that excellent performance of Cu-PPy-FMWCNTS nanocomposite is due to uniformly distributed Cu nanostructures and large internal surface area which service the reduction and diffusion of triiodide (I_3_^−^) throughout the nanocomposite film^63^. Therefore, it is concluded, that the novel Cu-PPy-FMWCNTS nanocomposite is a favorable alternative CE to replace the Pt CE.

## Conclusions

In conclusion, flexible Cu-PPy-FMWCNTS nanocomposites films were fabricated by simple two step electrodeposition method on SS substrate and were used as an efficient catalyst CE for reduction of triiodide ions in DSSCs. A DSSC with Cu-PPy-FMWCNTS nanocomposites CE achieves a photovoltaic efficiency of 7.1% which is comparable to the efficiency of solar cell based on traditional Pt CE. Higher photoelectric properties of Cu-PPy-FMWCNTS nanocomposites CE is due to its higher electrical conductivity (280 s/cm), better electrocatalytic ability and lower charge transfer resistance (4.31Ω/cm^2^), for I_3_^−^/I^−^redox reaction. The simple fabrication method and low cost of the Cu-PPy-FMWCNTS CE appears to be a potential substitute to the high-cost Pt for practical application of DSSCs.
